# Turning off the tap: sustaining elimination of congenital syphilis through the programme targeting high-risk groups

**DOI:** 10.7189/jogh.09.020312

**Published:** 2019-12

**Authors:** Xiang-Sheng Chen

**Affiliations:** 1 Institute of Dermatology, Chinese Academy of Medical Sciences (CAMS) & Peking Union Medical College (PUMC), Nanjing, China; 2 National Center for STD Control, Chinese Center for Disease Control and Prevention, Nanjing, China

Syphilis continues to affect a large number of pregnant women, causing substantial perinatal morbidity and mortality. It was estimated that approximately 1.36 million pregnant women globally were infected with syphilis, resulting in at least 520 000 adverse pregnancy outcomes caused by mother-to-child transmission (MTCT) of the infection [1]. As one of the adverse pregnancy outcomes of MTCT of syphilis, congenital syphilis can be prevented by screening and treatment of infected pregnant women. In light of continuing mortality caused by maternal syphilis and the high cost-effectiveness of antenatal screening and treatment for syphilis as an intervention strategy, the WHO launched the global initiative for the elimination of congenital syphilis in 2007 [2]. Since then, Cuba become the first country to be officially validated by the WHO for the successful elimination of congenital syphilis in 2015, followed by another 10 countries and territories by the end of 2018, including Belarus, Moldova and Thailand in 2016; Anguilla, Antigua & Barbuda, Bermuda, Cayman Islands, Montserrat, St. Christopher & Nevis in 2017 and Malaysia in 2018. In addition, more countries have had their validation plans in place or committed to applying for the validation by a target date. These achievements are primarily owing to integrating interventions of maternal syphilis into the existing elimination of MTCT of HIV. However, maternal syphilis in these countries which have achieved the elimination account for only a small part of the global burden of the disease. Many countries, particularly those with high burden of syphilis among pregnant women as well as those with high rate of the infection among high-risk groups, still face many challenges towards achieving the elimination of congenital syphilis. These challenges could be related to policy and resource, social environment, health system, programme implementation, and others in many resource-limited settings (**Box 1**).

Box 1 **Potential barriers to PMTCT of syphilis in many settings with high burden of maternal syphilis**

**Policy and resource**

• No policy on universal antenatal screening for syphilis;

• Inadequate fund allocation;

• Lack of coordination among stakeholders; and

• Lack of integration with maternal and child health programmes.

**Socio environment**

• Stigma from family members and community;

• Violence from sexual partners; and

• Negative traditional/religious beliefs.

**Health system**

• Poor health care system;

• Poor infrastructure;

• Inadequate human resources/trained staff; and

• Unavailability or stock-out of supplies (drugs, testing kits).

**Programme management**

• Poor management system;

• Lack of proper monitoring; and

• Lack of support and supervision.

**Others**

**Figure 1:**
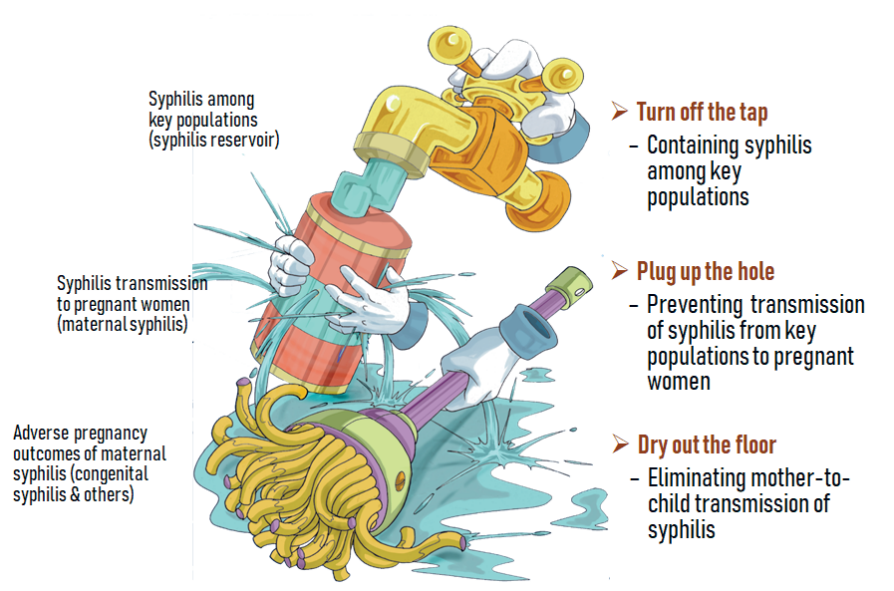
Strategies to eliminate transmission of syphilis from high-risk groups to pregnant women and from mother to baby.

Current version of the global initiative for elimination of MTCT (EMTCT) of syphilis emphasizes the coverages of intervention services targeting pregnant women, ie, ≥95% antenatal care (at least one visit), ≥95% syphilis testing and ≥95% treatment coverage (at least one dose of intramuscular benzathine benzylpenicillin) for at least 2 years in order to achieve the goal of having a congenital syphilis rate ≤50 cases per 100 000 live births. Increase of political commitment and financial investment from country governments and international donors, and early detection of infection by introduction of point-of-care (POC) tests in the first and second trimesters followed by treatment with benzathine penicillin have significantly facilitated the progress ensure the achievement of these intervention targets [3]. Special efforts to eliminate MTCT of syphilis together with EMTCT of HIV as well as hepatitis B have been made by increasing number of countries along with a continuing global and local advocacy particularly related to contribution of the elimination to progress towards the Sustainable Development Goals (SDGs), or partly because of the country’s interest in being certified by WHO for their achievement of this elimination. With these efforts, it may be possible for a country to achieve the validation targets within an anticipated frame of time through a transient campaign with increased government commitment and/or donor’s investment. However, these efforts can only be considered as “drying out the water” on the floor in the dynamics shown in **Figure 1**. To sustainably dry out the water may be a great challenge if control of the infection among the high-risk populations, such as men who have sex with men (MSM), female sex workers (FSWs), and injection drug users (IDUs), are neglected and the corresponding programs and necessary investments are not ensured (the tap is not turned off). A modeling study using the data in the WHO Global Health Observatory for nine African countries and the different scenarios indicates that even if all three process targets set by the global EMTCT initiative are met, none of these countries would achieve the goal of [4]. Lessons learnt from these countries indicate that elimination of congenital syphilis relies not just upon the guarantee of delivering these interventions targeting pregnant women, but most importantly on the effective control of syphilis among high-risk groups as well.

**Figure:**
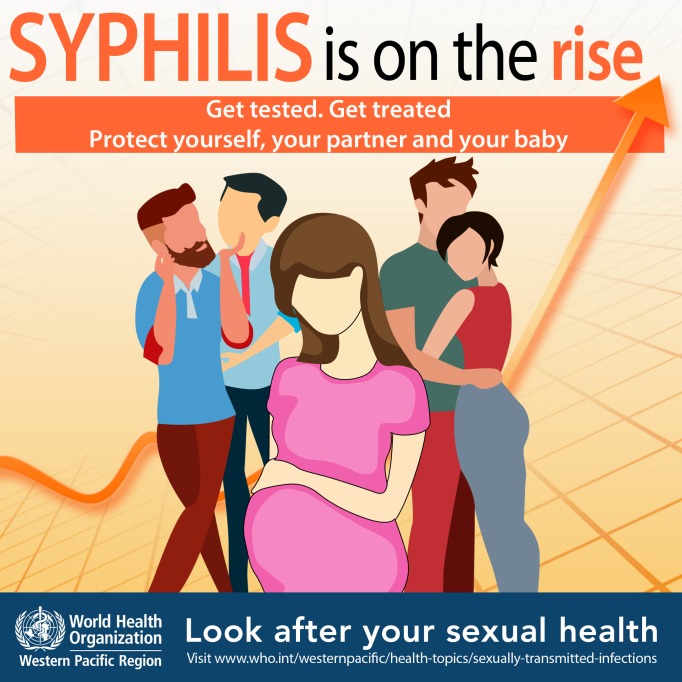
Photo: Syphilis in high-risk groups endangers pregnant women (from the World Health Organization, used with permission).

Sexually transmitted infections (STIs) other than HIV are usually neglected by many countries as a public health priority. Over the last two decades, the United States and China are the countries with national program specifically designed for control of syphilis although some of other countries initiated specific projects at national or subnational level, such as Attack of the Cursed Syphilis campaign to increase syphilis awareness in Toronto, and the National Gay Men's Syphilis Action Plan (NGMSAP) to increase syphilis testing among MSM at high risk in Australia [5]. The United States is the first country to release the national program by launching the National Campaign to Eliminate Syphilis from the United States, known as the elimination plan, with interim targets for syphilis elimination in 1999 [6]. Based on the lessons learned from implementing the plan and the fact of rising syphilis rate among MSM, the plan was revised to set up the new national targets and update the strategies as the 3-by-3 approach to syphilis elimination [7]. China is probably the only country to simultaneously release the specific national programs to address elimination of congenital syphilis and control of syphilis among high-risk groups. To address the epidemic, the Chinese Ministry of Health officially launched a national program directly aimed at control of syphilis - the National Program for Prevention and Control of Syphilis in China (2010-2020) in 2010 [8]. Beyond continuously promoting sustained behaviour change and condom promotion, interventions focusing on the Three Screenings Linked to One Standardized Care (3 × 1 SLSC) (**Box 2**) has been implemented as core strategies for syphilis control in China [8,9]. In 2011, the Ministry further released a national program specifically aimed at preventing PMTCT of HIV, syphilis and hepatitis B [10]. For implementation of these two parallel programmes, the central government tagged annually more than $140 million specially allocated for syphilis control as well as for the triple prevention of MTCT of HIV, syphilis and hepatitis B [8,10]. The data from the national surveillance program have indicated a turning point towards decreased incidence of primary and secondary (P&S) syphilis (usually defined as active syphilis) appeared in 2012 and one year later the turning points for congenital syphilis occurred, probably implying the impacts of syphilis control in high-risk groups on decline of congenital syphilis. At the moment, China has not yet been validated for EMTCT of syphilis and is still listed as one of the countries having had their plans in place for the validation by a target date. It is quite sure that the national program designed for interventions among high-risk groups will definitely provide strong guarantee to facilitate and sustain the elimination of congenital syphilis in China. Lessons learned from China may valuable for the prospective plan for achieving and sustaining EMTCT of syphilis at global and country level. In conclusion, elimination of MTCT of syphilis globally may be achievable but hard to be sustainable if the high rate of syphilis among high-risk groups is not well contained in addition to promoting the integration of maternal syphilis screening and treatment as part of basic antenatal health services [11].

Box 2 **Three screenings linked to one standardized care strategies**

**Three screenings**

• Providing an active syphilis screening to patients at risk for sexually transmitted infections (STIs) at clinics where STI service is delivered.

• Providing an active syphilis screening to clients who attend HIV voluntary counselling and testing (VCT) or methadone maintenance treatment (MMT) services followed by referral of syphilis-positives to STI clinics.

• Providing an active syphilis screening as part of outreach services targeting high-risk groups and referral of syphilis-positives to STI clinics.

**One standardized care**

• Providing syphilis-infected patients with a standardized care in which treatment with at least one shot of benzathine benzyl penicillin (BBP) should be ensured, and behavioural interventions, and partner notification be delivered.
